# Intracranial ependymoma: A retrospective analysis of clinical features, treatment modalities, and long‐term outcome

**DOI:** 10.1002/agm2.12378

**Published:** 2024-12-11

**Authors:** Amr Badary, Sarah Zuhair Kurdi, Yasser F. Almealawy, Sura N. Alrubaye, Vivek Sanker, Bipin Chaurasia, Oday Atallah

**Affiliations:** ^1^ Department of Neurosurgery SRH Wald‐Klinikum Gera Gera Germany; ^2^ Department of Neurosurgery University of Kufa Kufa Iraq; ^3^ Faculty of Medicine University of Kufa Kufa Iraq; ^4^ Faculty of Medicine University of Babylon Hilla Iraq; ^5^ Department of Neurosurgery Trivandrum Medical College Kerala India; ^6^ Department of Neurosurgery Neurosurgery Clinic Birgunj Nepal; ^7^ Department of Neurosurgery Hannover Medical School Hannover Germany

**Keywords:** ependymoma, intracranial, intraventricular, recurrence, WHO grade

## Abstract

**Objective:**

Ependymomas, rare neuroglial tumors originating from ependymal cells, can occur in the CNS and typically affect the brain's ventricles or spinal cord. Prognosis is influenced by tumor grade, location, resection extent, and preoperative Karnofsky Performance Status Scale (KPSS) scores. This study evaluates clinical features, treatment outcomes, and factors affecting prognosis in patients with intracranial ependymomas.

**Methods:**

A retrospective review of 23 patients with intracranial ependymomas, treated from 2018 to 2023, was conducted. Data included demographics, clinical presentations, KPSS scores, imaging findings, and treatment details. Outcomes assessed were postoperative complications, recurrence rates, and functional status. Statistical analysis used SPSS version 26, with significance set at *p* < 0.05.

**Results:**

The cohort was predominantly male (87.0%), with a mean age of 27 years. Tumors were mostly in the fourth ventricle (82.6%), with an average diameter of 68.9 mm. Complete resection was achieved in 87.0% of cases. Postoperative radiotherapy was given to 91.0% of grade 2 and all grade 3 tumors. Recurrence occurred in 17.4% of grade 2 ependymomas, but none of grade 3. The seven‐month mortality rate was 4.3%. Higher preoperative KPSS scores correlated with better outcomes.

**Conclusion:**

Complete tumor resection and postoperative radiotherapy are crucial for improved outcomes in ependymomas. Higher preoperative KPSS scores and tumor location significantly impact prognosis. Tumors in the lateral ventricles are associated with higher recurrence risks. These findings highlight the need for aggressive surgical management and personalized adjuvant therapy to enhance patient outcomes.

## Graphical abstract

In adults, ependymomas are more frequently discovered in the spine; in children, they are usually located in the brain. The standard course of treatment for ependymomas is surgery. Image showing an overview of intracranial ependymomas.

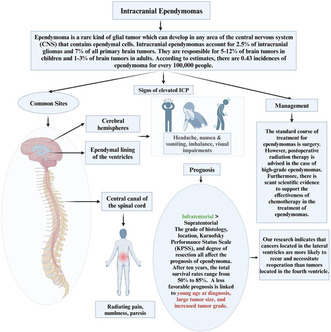

## 1 INTRODUCTION

Ependymoma is an uncommon type of glial tumor that can occur in any part of the central nervous system (CNS) where ependymal cells are present.^1^ The typical locations of their genesis are the central canal of the spinal cord, the cerebral hemispheres, and the ependymal lining of the ventricles.^2^, ^3^ Juvenile ependymomas are predominantly located in the brain, especially the posterior fossa, but spinal tumors are more prevalent in adults.^4^

Several investigations have established a correlation between the occurrence of ependymomas in individuals with NF2.^4^ In the yearly assessment of primary central nervous system tumors, intracranial ependymomas constitute 2.5% of all intracranial glial cell tumors and 7% of all primary brain tumors.^3^ They account for 1%–3% of brain tumors in adults and 5%–12% of brain tumors in children. The incidence of ependymoma is estimated to be 0.43 cases per 100,000 individuals.^3^

Surgery is the typical treatment for ependymomas. Nevertheless, it is recommended to have postoperative radiation therapy for high‐grade ependymomas. Moreover, there is limited empirical support for the efficacy of chemotherapy in treating ependymomas.^5^ The prognosis of ependymoma is influenced by various parameters, such as the grade of histology, location, Karnofsky Performance Status Scale (KPSS), and the extent of resection. The overall survival rates at 10 years vary between 50% and 85%.^4^ Patients diagnosed with infratentorial ependymomas often have a somewhat more favorable prognosis in comparison to those with supratentorial ependymomas.^6^, ^7^ Youthful age at diagnosis, substantial tumor size, and elevated tumor grade are associated with a less favorable prognosis.^3^

The purpose of this retrospective analysis is to assess factors such as clinical features, pathology, imaging, post‐surgery complications, tumor recurrence, and functional outcomes within this specific cohort.

## 2 METHODS

### 2.1 Eligible patients

This retrospective study included 23 patients diagnosed with intracranial ependymoma from 2018 to 2023. Patients were eligible for inclusion if they had undergone either complete or partial tumor excision, with or without additional radiotherapy or chemotherapy. To maintain the accuracy and reliability of the findings, cases with incomplete or insufficiently detailed medical records were excluded from the study. All eligible patients were required to have detailed preoperative imaging, clinical evaluations, and follow‐up data available for analysis.

### 2.2 Data collection

Data collected from patient medical records included demographic details (such as age and gender), clinical presentations (including symptoms like headaches, vomiting, and seizures), comorbid conditions, and preoperative Karnofsky Performance Status Scale (KPSS) scores. Imaging data were reviewed to document tumor characteristics, such as location, size, and the presence of hydrocephalus. Treatment details were recorded, including the type of surgical procedure performed, use of external ventricular drainage (EVD), shunting procedures, and any adjuvant therapies administered.

### 2.3 Treatment protocols

All patients underwent surgical resection as part of their treatment. Surgical procedures varied depending on the individual case, with some patients receiving complete resection and others undergoing partial resection. Additional treatments, such as radiotherapy and chemotherapy, were administered based on tumor characteristics and patient condition. The use of EVD and shunting procedures was documented when required to manage hydrocephalus.

### 2.4 Outcome measures

Outcomes evaluated included postoperative complications, mortality rates, tumor recurrence, and functional status as measured by KPSS at both discharge and final follow‐up. Patients underwent routine imaging, including MRI of the brain, to assess tumor status and monitor for recurrence. Functional status assessments and evaluations for any postoperative complications were performed at regular intervals.

### 2.5 Statistical analysis

Statistical analyses were conducted using SPSS version 26 (IBM Corp., Armonk, NY, USA). Continuous variables were summarized using means and standard deviations or medians and interquartile ranges, depending on the data distribution. Categorical variables were summarized using frequencies and percentages. For comparisons between groups, independent sample *t*‐tests or Mann–Whitney U tests were used for continuous variables, while chi‐square tests or Fisher's exact tests were applied for categorical variables.

To control for multiple comparisons and manage the family‐wise error rate, the Bonferroni correction was applied. This method involved calculating adjusted *p*‐values for analyses involving multiple outcome measures to maintain statistical rigor. After adjustment, a *p*‐value of <0.050 was considered statistically significant. Crosstabulation was conducted to assess the independent effects of preoperative KPSS scores, tumor location, and extent of resection on overall survival. Although multivariate analysis was initially considered, it was not performed due to the small sample size, which posed a high risk of bias.

## 3 RESULTS

The study included patients diagnosed with intracranial ependymoma, regardless of age, who received either complete or partial tumor resection, with or without following radio‐chemotherapy. The patients in this study were monitored starting from the moment of their surgery for an average duration of 34 months after the operation, with a standard deviation of 14 months (Table 1).

**TABLE 1:** Showing the patients characteristics and some of the radiological findings.

*n*	Age	Gender	H/A	Seizures	Vomiting	PreOP KPSS	HCP	Site	Tumor size	Comorbidities
1	6	Male	Yes	No	Yes	100	Yes	4th V	23 × 50 mm	No
2	24	Male	Yes	No	No	90	No	4th V	56 × 70	No
3	10	Male	Yes	No	Yes	90	Yes	4th V	34 × 50	Asthma
4	45	Male	Yes	No	No	90	Yes	4th V	62 × 43	No
5	13	Male	Yes	No	Yes	90	Yes	4th V	80 × 67	No
6	6	Male	Yes	No	Yes	90	Yes	4th V	64 × 50	No
7	28	Female	Yes	No	No	90	No	4th V	19 × 43	No
8	50	Male	Yes	Yes	No	90	No	Lateral v	98 × 80	HTN
9	12	Male	Yes	No	Yes	90	Yes	4th V	37 × 56	NF2
10	23	Male	Yes	No	No	90	Yes	4th V	70 × 30	No
11	20	Male	Yes	No	No	90	Yes	Lateral v	46 × 63	No
12	35	Male	Yes	No	No	90	Yes	4th V	56 × 80	No
13	48	Male	Yes	No	No	80	Yes	Lateral v	90 × 77	No
14	32	Female	Yes	No	No	90	Yes	4th V	70 × 62	No
15	45	Male	Yes	No	No	90	Yes	4th V	28 × 56	No
16	30	Male	Yes	Yes	No	90	Yes	Lateral v	36 × 56	No
17	26	Male	Yes	No	No	90	No	4th V	70 × 64	No
18	18	Female	Yes	No	Yes	90	Yes	4th V	55 × 78	No
19	40	Male	Yes	No	No	80	Yes	4th V	35 × 60	No
20	33	Male	Yes	No	No	90	Yes	4th V	72 × 45	No
21	36	Male	Yes	No	No	90	Yes	4th V	45 × 60	No
22	26	Male	Yes	No	No	70	Yes	4th V	80 × 58	No
23	15	Male	Yes	No	Yes	90	Yes	Lateral v	92 × 75	No

**FIGURE 1:**
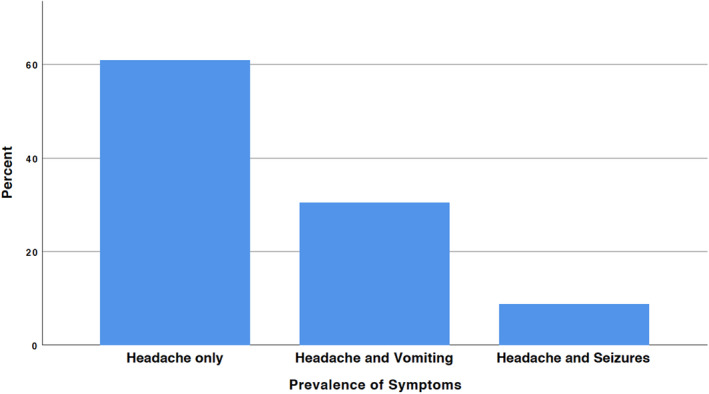
The prevalence of the associated symptoms.

Out of the 23 patients, 20 patients (87%) were male, while 3 patients (13%) were female. The average age of the patients was 27 years, with a standard deviation of 13 years and a 1‐year age difference between genders. All patients universally reported headaches (100%), while 6 individuals experienced vomiting (30%), and 2 individuals had seizures (10.0%) (Figure 1). In addition, three individuals had simultaneous comorbidities: arterial hypertension, asthma, and neurofibromatosis. Prior to the surgery, the average score on the KPSS was 88.7%, with a standard deviation of 5.4%.

Radiological assessment revealed that the average longest diameter of the tumors was 68.9 mm, with a standard deviation of 14.0 mm. The median tumor volume was 114 cm^3^, with specific median volumes of 92.8 cm^3^ for tumors associated with the 4th ventricle and 251.98 cm^3^ for those associated with the lateral ventricle. Tumors were predominantly located in the fourth ventricle (82.6%) or the lateral ventricle (17.4%). Additionally, 19 patients (82.6%) experienced hydrocephalus, while 4 patients (17.4%) did not. No statistically significant associations were found between tumor location or size and the presence of hydrocephalus (*p*‐value = 0.142).

Out of the group, 20 patients (87%) had their tumors completely removed, while 3 patients (13%) had only a partial removal. Among these, 16 patients underwent EVD followed by shunting. Out of these, 15 patients had hydrocephalus, accounting for 79% of those with hydrocephalus, while 1 patient, which represents 25.0% of those without preoperative hydrocephalus, did not have hydrocephalus. A single patient who did not have hydrocephalus before surgery had EVD and shunting due to the recurrence. Significantly, four patients with hydrocephalus experienced improvement without requiring EVD or shunting. There was a single occurrence of postoperative complications in one patient each for CSF leak and facial palsy, accounting for a complication rate of 8.7% (4.3% each). Recurrence was also seen by 3 patients (13% of the total), and reoperation was necessary in these cases (Table 2).

**TABLE 2:** Showing the management and the outcomes.

*n*	TTR	EVD	Comp	RT	Chemoth	Append, Grade	Rec	Re‐OP	Shunt	Outcome at discharge	Mort	Follow up	KPSS at last follow‐up	PFS (M)
1	Yes	Yes	No	Yes	Yes	G2	No	No	Yes	Good	No	24	90	15
2	Yes	No	No	No	No	G2	No	No	No	Good	No	36	90	10
3	Yes	Yes	No	Yes	No	G2	No	No	Yes	Good	No	24	90	12
4	Yes	Yes	No	Yes	No	G2	No	No	Yes	Good	No	36	90	10
5	Yes	Yes	No	Yes	No	G2	Yes	Yes	Yes	Fair	No	50	80	20
6	Yes	Yes	CSF leak	Yes	Yes	G2	No	No	Yes	Good	No	6	90	15
7	Yes	No	No	Yes	No	G2	No	No	No	Good	No	19	90	15
8	No	No	No	Yes	No	G2	No	No	No	Good	No	33	90	20
9	Yes	Yes	No	Yes	No	G2	No	No	Yes	Good	No	50	90	20
10	Yes	Yes	No	Yes	No	G2	No	No	Yes	Good	No	38	90	20
11	Yes	No	No	Yes	No	G2	Yes	Yes	No	Good	No	46	90	20
12	Yes	Yes	No	Yes	No	G2	No	No	Yes	Good	No	27	90	20
13	No	Yes	Facial Palsy	Yes	No	G3	No	No	Yes	Fair	No	50	70	18
14	Yes	Yes	No	Yes	No	G2	No	No	Yes	Good	No	25	90	6
15	Yes	Yes	No	Yes	No	G2	No	No	Yes	Good	No	15	90	4,5
16	Yes	No	No	Yes	No	G2	No	No	No	Good	No	37	90	8
17	Yes	Yes	No	Yes	No	G2	Yes	No	Yes	Good	No	48	90	18
18	Yes	Yes	No	No	No	G2	No	No	Yes	Good	No	45	90	14
19	No	Yes	No	Yes	No	G2	No	No	Yes	Poor	7 m	7	50	21
20	Yes	Yes	No	Yes	No	G2	No	No	Yes	Good	No	18	90	11
21	Yes	No	No	Yes	No	G2	No	No	No	Good	No	44	90	9
22	Yes	Yes	No	Yes	No	G3	No	No	Yes	Poor	No	51	60	7
23	Yes	No	No	Yes	No	G2	Yes	Yes	No	Good	No	53	90	11

**FIGURE 2:**
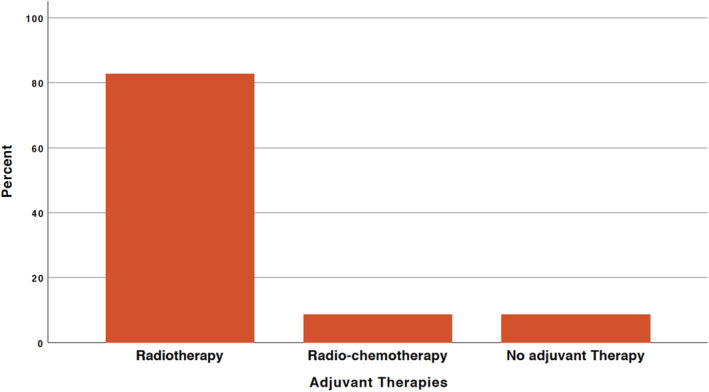
The adjuvant therapies used across the cohort.

In relation to further treatments for ependymoma grades 2 and 3, 21 patients (91%) and 2 patients, respectively (9%) (Figure 2), 81.0% of the 21 patients with grade 2 received only postoperative radiotherapy, 9.5% had radio‐chemotherapy, and 9.5% did not get any postoperative adjuvant therapy. Postoperative radiation was administered entirely to all patients with grade 3 (100%). Out of the three patients (13%) who had a partial removal of the tumor, one had grade 3 and two had grade 2. These patients had postoperative radiotherapy as their only additional treatment.

**FIGURE 3:**
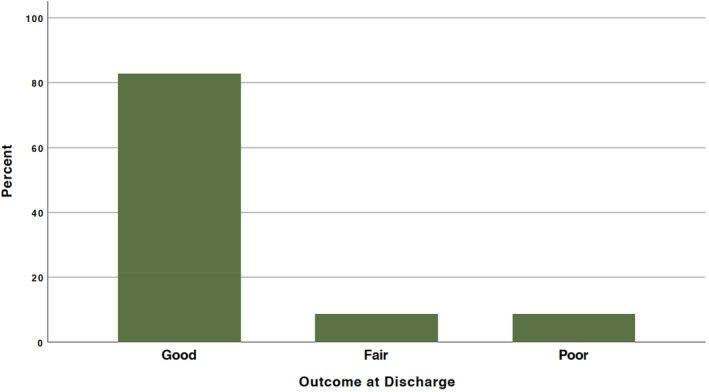
The different outcomes in our study.

The outcome of the analysis showed that 19 patients (82.6%) had good outcomes, whereas 2 patients (8.7%) had fair outcomes, and 2 patients (8.7%) experienced poor outcomes (Figure 3). Unfortunately, the mortality rate in the seventh month after surgery was only 4.3%, with only one patient succumbing. Recurrence occurred in 4 out of 23 patients (17.4%) diagnosed with ependymoma grade 2; however, none of the 2 patients (0/2, 0%) with grade 3 experienced recurrence. The mean KPSS score at follow‐up was 85%, with a standard deviation of 10.7%. The death rate at the last follow‐up, which occurred on average 14 months after the study began, was 4.3%.

Patients with a preoperative KPSS of 90% or higher generally experienced better outcomes compared to those with a KPSS below 90%. They had lower death rates, more favorable KPSS scores upon discharge, and overall better results (*P*‐value = 0.039). Our study found that patients with ependymomas placed in the lateral ventricles have a higher likelihood of having recurrence and requiring reoperation compared to those with ependymomas in the fourth ventricle (*P*‐value = 0.047). In addition, there was no effect of the tumor volume on the preoperative or postoperative KPSS, whether the mass was related to the 4th ventricle or the lateral ventricle.

Based on statistical analysis, those who had a complete removal of the tumor often have greater outcomes when they are discharged from the hospital. They also have a decreased chance of dying and show improved scores on the KPSS at their last follow‐up, compared to those who had a partial removal of the tumor (*P*‐value = 0.041).

To assess the impact of follow‐up duration on our outcomes, we adjusted for this variable using Cox proportional hazards models. This analysis accounted for the varying lengths of follow‐up and provided a more precise estimation of hazard ratios for recurrence and progression. The adjusted model showed that the hazard ratio for recurrence was 1.054 (95% CI: 0.982–1.124) per additional month of follow‐up, and the hazard ratio for progression‐free survival was 0.972 (95% CI: 0.903–1.051). These findings indicate that follow‐up duration slightly influences risk but does not significantly alter the overall conclusions of our study.

## 4 DISCUSSION

In 1863, the German pathologist Rudolf Virchow, often regarded as the “father of modern pathology,” first identified rosette‐like clusters of ependymal cells in brain tumors and named these tumors “ependymomas.”^8^ Ependymomas are a heterogeneous group of neuroectodermal neoplasms that originate from glial cells and can be found in various locations along the neuroaxis, including the cerebral ventricles, choroid plexus, central canal of the medulla, spinal cord, and filum terminale.^9^ They may arise from residual fetal ependymal cells throughout brain development.^3^, ^10^ Despite advances, the precise genetic and molecular features of ependymomas remain incompletely understood.^9^

### 4.1 Demographics and prevalence

Ependymomas constitute 2% to 8% of all primary CNS tumors, with a prevalence of up to 12% in pediatric cases.^11^ They are the second most common CNS neoplasm in children and adolescents, following medulloblastomas.^11^ While brain tumors account for 5%–12% of pediatric cases, they represent only 1%–3% of CNS tumors in adults.^3^, ^12^, ^13^ In the United States, the age‐adjusted annual incidence rate of ependymomas is 0.41 per 100,000, and 0.20 per 100,000 in Europe.^3^, ^9^, ^10^, ^14^ The tumors are more prevalent in males compared to females by 10%–15%, more common in white populations than in other ethnic groups, and more frequent in non‐Hispanic individuals than in Hispanics.^15^ Our findings align with this, showing that 87% of affected individuals were male, and a higher mortality rate, though less frequent, was noted in the African American community compared to whites.^15^

Ependymomas demonstrate a bimodal age distribution, peaking at ages 3 and 57, with the highest incidence between 45 and 64 years.^2^, ^9^ Tumor location varies by age group: in adults, they are more commonly found in the spine, while in children, they are often located in the brain, particularly in the posterior fossa.^2^ The mean age of our patient cohort was 27 years, reflecting the bimodal distribution of ependymomas with peaks in early childhood and late adulthood.^2^, ^9^ The only well‐established risk factor for ependymomas is neurofibromatosis type 2 (NF2), which predisposes individuals to these tumors.^2^, ^16^ NF2‐associated ependymomas typically exhibit delayed growth and more benign behavior.^16^ In our study, only one patient had NF2.

### 4.2 Pathophysiology

Ependymomas are typically characterized by rapid growth, with more than 90% exceeding a diameter of 4 cm.^17^, ^18^ In our study, the average longest diameter of the tumors was 68.95 mm. The majority of tumors were situated in the fourth ventricle (82.6%), while the remainder were in the lateral ventricle (17.4%). The median tumor volume was 114.46 cm^3^, with specific median volumes of 92.82 cm^3^ for tumors in the 4th ventricle and 251.98 cm^3^ for those in the lateral ventricle.

### 4.3 Clinical presentation

**FIGURE 4:**
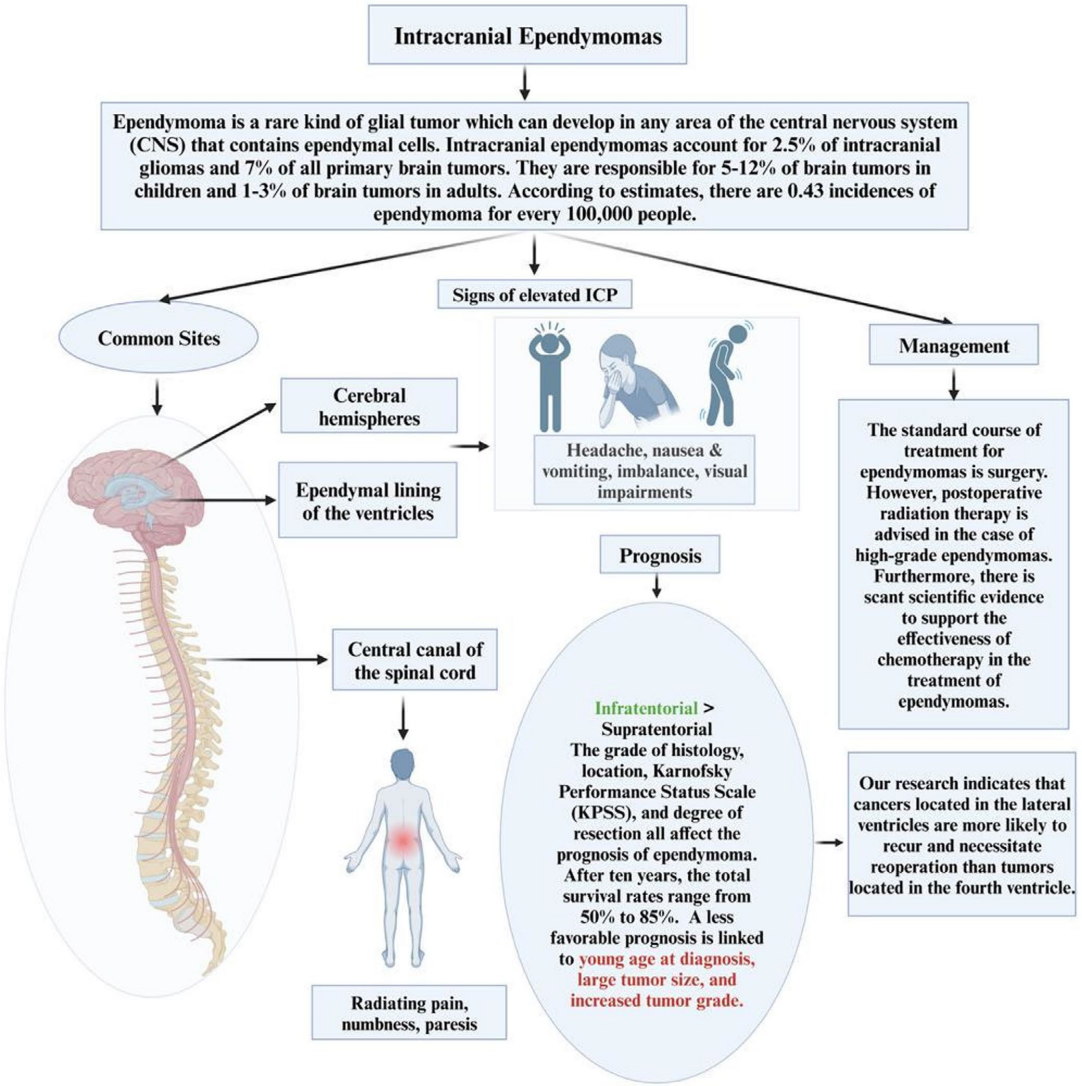
An overview of intracranial ependymomas.

Clinically, ependymomas present differently depending on age and tumor location. Symptoms may be absent for extended periods, leading to late diagnosis.^19^ Tumors in the fourth ventricle often present with signs of intracranial hypertension such as fatigue, headaches, vomiting, nausea, balance issues, and visual impairments.^2^, ^19^, ^20^, ^21^ These cases frequently involve multiple cranial nerve palsies and cerebellar dysfunction due to the tumor's proximity to the fourth ventricle. Children with posterior fossa ependymomas often show symptoms related to obstructive hydrocephalus.^2^, ^19^, ^20^, ^21^ Our study corroborates these findings, with all patients experiencing headaches, 30% experiencing vomiting, and 10% having seizures (Figure 4).

### 4.4 Diagnostic evaluation

Imaging is crucial for diagnosing ependymomas prior to treatment.^22^, ^23^ Ependymomas typically present as well‐defined lesions with varying contrast enhancement. They may also show calcifications, bleeding, and surrounding edema.^4^, ^23^ MRI is the preferred diagnostic tool, providing detailed information on tumor location, shape, and internal composition, with ependymomas usually appearing as well‐defined masses with low T1 signal, high T2 signal, and intermediate to high FLAIR signal.^4^, ^23^ Occasionally, they may show calcifications, bleeding, necrosis, and cystic formations, especially in supratentorial lesions.^4^ Contrast‐enhanced CT scans can also show heterogeneous enhancement, though less detailed.^22^ Additionally, our study found that patients with ependymomas in the lateral ventricles had a higher likelihood of recurrence and the need for reoperation compared to those with tumors in the fourth ventricle (*p*‐value = 0.047). This suggests that tumor location may affect recurrence risk, consistent with previous research.^24^

### 4.5 Management

Surgical resection remains the primary treatment for ependymomas, with adjuvant therapies such as radiation and chemotherapy applied based on tumor characteristics.^25^ Our results showed that of 20 patients, (87%) had complete tumor removal, and 3 patients (13%) had partial removal. Among those who had partial resections, 18 patients (79%) had hydrocephalus, and EVD followed by shunting was performed in 16 patients. Notably, 4 patients with hydrocephalus improved without requiring EVD or shunting. This is consistent with the literature, which indicates that complete tumor removal is linked to improved outcomes and reduced recurrence rates.^26^, ^27^ While radical resection is the most effective treatment approach, additional therapies, such as radiotherapy, may be required for cases of incomplete resection or anaplastic tumors.^24^, ^28^ Our study found a complication rate of 8.7%, with CSF leaks and facial palsy each occurring in 4.3% of patients, and 13% experienced tumor recurrence requiring reoperation. Intraoperative neuro‐monitoring is crucial for complete removal, especially when tumors are attached to the floor of the fourth ventricle.^27^ Ependymomas present surgical challenges due to their location and tendency for incomplete removal.

Pathological examination confirms ependymoma diagnosis by identifying differentiated ependymal cells and distinct histopathological features such as perivascular pseudorosettes and true ependymal rosettes.^29^ Histologically, ependymomas often have round‐to‐oval cells with speckled chromatin.^13^, ^29^ The variability in histological appearance can make accurate diagnosis challenging. The WHO 2021 classification integrates molecular and histological traits, categorizing ependymomas into 10 subgroups and improving diagnostic accuracy.^13^ However, predicting patient outcomes based solely on histological or molecular markers, especially distinguishing RELA fusion‐positive (II/III) and anaplastic (III) ependymomas, remains complex.^13^, ^30^ Our study found that 21 patients (91%) had WHO grade II ependymoma.

In a multicenter study of treating patients with recurrent ependymomas, maximal safe resection was identified as a key factor for improved long‐term survival.^31^ The study found that Temozolomide offered limited benefit as a first‐line chemotherapy, with a partial remission rate of only 14.3%.^31^ Radiotherapy was beneficial primarily in cases with subtotal resection or no prior surgery, although its effectiveness varied and depended on the extent of previous treatments.^31^

### 4.6 Postoperative care and follow‐up

Performance status is a key predictor of survival, with lower scores linked to worse outcomes. Incorporating performance status into clinical trials can improve study design and survival predictions, especially when combined with other clinical factors.^32^ Evidence suggests that patients with a preoperative Karnofsky Performance Status Score (KPSS) of ≤60% may experience notable benefits from surgical resection.^33^ Postoperative KPSS scores are reliable for predicting survival and can assist in risk stratification and patient counseling.^34^ Further validation in additional cohorts is recommended.^34^ Our analysis showed that 19 patients (82.6%) had good outcomes, 8.7% (2 patients) had fair outcomes, and another 8.7% (2 patients) had poor outcomes. The seven‐month postoperative mortality rate was 4.3%, with only one patient deceased. Patients with a preoperative KPSS of 90% or higher generally had better outcomes, lower mortality rates, and more favorable KPSS scores at discharge (*p*‐value = 0.039), supporting the association between better preoperative performance status and improved outcomes.^32^, ^33^

Postoperative management is essential for favorable outcomes in ependymoma surgery. Potential complications include infection, cerebrospinal fluid leaks, cranial nerve palsies, and shunt‐dependent hydrocephalus.^26^, ^27^ Shunting decisions depend on factors such as preoperative hydrocephalus and tumor characteristics.^35^ About 18% of patients with fourth‐ventricle ependymomas require shunting.^27^ Our study found a complication rate of 8.7%, with CSF leaks and facial palsy each occurring in 4.3% of patients, and 13% experienced tumor recurrence requiring reoperation.

Postoperative radiotherapy (RT) after partial resection can extend survival without disease progression.^26^, ^36^, ^37^ RT helps lower the risk of recurrence, reducing the need for additional surgeries, which are often linked to substantial morbidities.^18^, ^36^, ^37^, ^38^, ^39^ In our study, 91% of patients with grade 2 ependymomas received postoperative radiotherapy, while 9% underwent radio‐chemotherapy. All patients with grade 3 ependymomas were treated with postoperative radiotherapy. These results align with existing literature, which supports the use of radiotherapy as a standard adjuvant treatment for higher‐grade ependymomas.^16^ Recurrence occurred in 17.4% of grade 2 patients, but no grade 3 patients experienced recurrence, underscoring the variability in recurrence risk associated with tumor grade.^26^, ^40^

Tumor recurrence likelihood is influenced by histological diagnosis and resection extent.^25^ Anaplastic ependymomas have a higher recurrence rate compared to lower‐grade types.^26^, ^40^ For grade 2 ependymomas, some patients receiving radiotherapy after incomplete resection showed no recurrence even after over 10 years.^26^ In our study, 19% of grade 2 patients experienced recurrence after surgical resection and postoperative radiotherapy, highlighting the need for accurate histological diagnosis and tailored treatment.

### 4.7 Limitations

A limitation of this study is the relatively small sample size, which may limit the statistical power to detect significant differences among subgroups and may increase the risk of Type II errors. The small sample size may also affect the generalizability of our findings to broader populations, particularly in heterogeneous conditions such as ependymomas, where genetic and molecular variations could influence outcomes. The range in follow‐up times among patients, from as little as 6 months to as many as 53 months, can introduce significant variability in outcome measures such as progression‐free survival (PFS) and recurrence rates. To account for this variability, we conducted a sensitivity analysis, which revealed that while the average follow‐up duration did affect the overall outcome metrics, the trend in the data remained consistent. This analysis reinforces the reliability of our findings despite the differences in follow‐up duration.

## 5 CONCLUSION

This study reaffirms the significance of complete tumor resection and postoperative radiotherapy in the management of ependymomas. Our findings demonstrate that patients with higher preoperative KPSS scores and those undergoing complete resection generally experience better outcomes, with lower mortality and recurrence rates. Tumor location is a critical factor, as ependymomas in the lateral ventricles are associated with higher recurrence and reoperation rates compared to those in the fourth ventricle. These results emphasize the necessity of tailored treatment strategies, including radical surgical approaches and targeted adjuvant therapies, to improve survival and functional outcomes in ependymoma patients. Further research with larger sample sizes and longer follow‐up periods is needed to validate these findings and refine treatment protocols.

## AUTHOR CONTRIBUTIONS

All authors contributed to the study conception and design. The study was conceptualized and supervised by A.M. and O.A. Material preparation, data collection, and analysis were performed by S.Z.K., Y.F.A., and S.N.A. The first draft of the manuscript was written by A.B. and S.Z.K., and all authors commented on previous versions of the manuscript. All authors read and approved the final manuscript.

## FUNDING INFORMATION

The following research received no external funding.

## CONFLICT OF INTEREST STATEMENT

The authors declare no conflicts of interest.

## ETHICS STATEMENT

In view of the retrospective nature of the study, formal approval of the institutional ethics committee was not required at the authors' institution (University of Kufa, Iraq).

## INFORMED CONSENT

All patients consented to the scientific use of their medical data.

## Data Availability

The data presented in the following study are available from the corresponding authors upon request.
